# Genetic Diversity and Population Structure in Bryophyte With Facultative Nannandry

**DOI:** 10.3389/fpls.2021.517547

**Published:** 2021-04-07

**Authors:** Annick S. Lang, Thies Gehrmann, Nils Cronberg

**Affiliations:** ^1^Department of Biology, Lund University, Lund, Sweden; ^2^Biomedical Data Sciences, Leiden University Medical Center, Leiden, Netherlands

**Keywords:** SNP, sexual reproduction, genetic variation, spore dispersal, nannandry, moss (Musci)

## Abstract

Among plants, gender dimorphism occurs in about 10% of all angiosperms and more than 50% of all moss taxa, with dwarf males (DM) found exclusively in some unisexual mosses. In this study, we explore the role of male dwarfism as a reproductive strategy in the widespread acrocarpous moss *Dicranum scoparium*, which has facultative male dwarfism, having both dwarf males (DMs) and normal-sized males (NMs). We retrieved 119 SNP markers from transcriptomes which were used to genotype 403 samples from 11 sites at seven localities in southern Sweden. Our aims were to compare the genetic variability and genetic structure of sexually reproducing populations at different geographic levels (cushion, site, and locality) and compare in particular the relative contribution of females, dwarf males and normal-sized males to the observed genetic diversity. The numbers of DMs differed strongly between sites, but when present, they usually outnumbered both females and NMs. Low genetic differentiation was found at locality level. Genetic differentiation was strongest between cushions for females and NMs and within cushions for DMs indicating small scale structuring and sometimes inbreeding. NMs were more clonal than either DMs or females. Genetic diversity was similar between females and DMs, but lower for NMs. Two haplotypes were shared between females and DMs and one haplotype was shared between a DM and a NM. In conclusion, our results show that DMs and NMs play different roles in reproduction, inbreeding may occur at cushion level, but gene flow is high enough to prevent substantial genetic drift.

## Introduction

Unisexuality is usually associated with a certain degree of sexual dimorphism (Badyaev, 2002; Poissant et al., 2010). Evolution of size dimorphism can occur when males and females have distinct roles in reproduction and potentially different requirements, so that they are exposed to contrasting natural and/or sexual selection pressures (Badyaev, 2002; Isaac, 2005). For example, sexual size dimorphism with larger males is generally attributed to combat ability, dominance establishment and access to females for mating (e.g., McElligott et al., 2001; Isaac, 2005) and is mostly found in mammals. Reversed sexual size dimorphism, with larger females, is found in many animals, particularly among invertebrates. It is explained as (1) a response of reduced male-male competition, (2) extreme niche differentiation between genders, or (3) may be the result of the “Ghiselin-Reiss small-male hypothesis” (Blanckenhorn et al., 1995), in which selection favor small males because they require less energy and hence can invest more time in sexual activities. Male dwarfism, a form of extreme size dimorphism where male measure at most half of the size of females, is most common in marine taxa (Fairbairn et al., 2007) and in small organisms, such as invertebrates, fishes, algae, and bryophytes. It might occur when (4) the probability to encounter a sexual partner is low and the need to keep sexual partners nearby is high, as for example in the bone-eating marine worm *Osedax* or anglerfishes (Vollrath, 1998; Pietsch, 2005; Vrijenhoek et al., 2008; Rouse et al., 2015): in these cases males evolved into rudimentary creatures, which are permanently attached to a normal-sized female. In contrast to well-known examples from the animal kingdom, the evolution of sexual dimorphism in plants has been much less studied (Geber et al., 1999; Puixeu et al., 2019), although gender dimorphism occur in approximately 6–10% of all angiosperms (Geber et al., 1999; Vamosi et al., 2003) and in more than half of all moss taxa (Vanderpoorten and Goffinet, 2009; Frey and Kürschner, 2011). Male dwarfism, also called nannandry, has evolved several times independently in unrelated moss families (Vanderpoorten and Goffinet, 2009; Hedenäs and Bisang, 2011) and can either be obligate or facultative, i.e., males growing only as dwarfs or occurring as normal-sized males as well.

Mosses and other bryophytes (liverworts and hornworts) share the trait of having a lifecycle dominated by the haploid gametophyte, the diploid sporophyte generation is initiated after sexual fertilization and remain connected to the maternal shoot throughout its short existence. Most mosses are capable of both asexual and sexual reproduction. Asexual reproduction is common in mosses, allowing clonal regeneration from gametophytic fragments or specialized vegetative diaspores (gemmae). Sexual reproduction depends on water for antherozoids (sperm cells) to swim to the archegonia (Cronberg et al., 2006a; Rydgren et al., 2006). Due to the water dependency, fertilization in unisexual moss species is hampered by drought and physical separation of males and females (Vanderpoorten and Goffinet, 2009; Hedenäs and Bisang, 2011). Asexual reproduction is significant for short-range colonization and maintenance of existing populations and genotypes (Laaka-Lindberg et al., 2003; Glime, 2007). Sexual reproduction is important for recombination during meiosis which takes place in the diploid sporophyte, for adaptability and maintenance of genetic diversity as well as for long-range dispersal through mostly wind-dispersed spores (Cronberg, 2000). A high realized gene flow is often assumed from molecular markers (Patiño and Vanderpoorten, 2018), but attempts to sow spores in natural environment have often failed (Mishler, 1988) and only few studies have been designed to actually reveal genetic variability and genetic population structures at local scales.

Nannandry is unique to mosses among land plants and appears to have evolved as a means to increase the fertilization efficiency by decreasing the separation distance between genders and thus increasing the number of available mating partners for a female (Moya-Laraño et al., 2002; Hedenäs and Bisang, 2011; Rosengren and Cronberg, 2014). Minute males, originating from wind-dispersed spores, grow epiphytically on a much larger female and remain attached during their (bi)annual life-cycle. The growth is reduced to a few leaves, but nevertheless they produce fertile sexual branches (perigonia). Several dwarf males can be found on a single female, usually clustering close to the female sexual branches (perichaetia). Whereas the gender is controlled by sex chromosomes, empirical studies have demonstrated that the growth form of males (dwarfed or normal-sized) could either be genetically determined (in species with obligate dwarf males) or controlled by the supporting female (Glime and Bisang, 2017 and reference therein).

If females in a nannandric species are predominantly fertilized by dwarf-males we can expect that the system is driven toward higher specialization for nannandry (e.g., smaller male spore sizes, with higher dispersal capacity at the expense of lower potential to germinate in the normal substrate) and eventually obligate nannandry. In a species with facultative nannandry, a dwarf male transmits its genes during one season, whereas a normal-sized male can transmit its genes over many years in the same patch and hence could slow down adaptation. However, if normal-sized males and dwarf-males are equally frequently fertilizing the females, the system may stay in a facultatively nannandric condition, without drift toward obligate nannandry. The relative frequency of dwarf males and normal-sized males and their associated haplotypic networks is therefore a key for understanding the development of a nannandric species.

Recent ecological and genetical studies of the nannandric species *Homalothecium lutescens* (Hedw.) H. Rob. have greatly extended our knowledge about nannandry in bryophytes (Rosengren et al., 2013, 2015, 2016). This species is pleurocarpous, having sporophytes on short lateral branches and forming loose and patchy colonies on calcareous ground. Recruitment of dwarf males takes place within colonies, sometimes leading to extreme inbreeding, but the studies also revealed that gene flow occurs between colonies within a population and even from external populations, frequently enough to maintain genetic variability and to prevent substantial genetic drift at population level. The fact that *H. lutescens* is almost completely obligately nannandrous has important implications for its population dynamics; dwarf males are sensitive to environmental factors such as drought, which may cause them to go extinct from time to time in local patches, while the females are more strongly clonal and persistent. This means that females follow a repeated recruitment model (sensu Eriksson, 1989, 1993) with perennial, highly clonal patches, whereas the DMs follow a metapopulation recruitment model (sensu Levins, 1969), having annual, non-persistent individuals with strong fluctuation in numbers between years.

The genus *Dicranum* is unrelated to *Homalothecium*, belonging to the acrocarpous moss groups, characterized by apical sporophytes on erect sparsely branched shoots and growth in dense tufts, radially expanding through vegetative proliferation. About half of the 131 *Dicranum* species reproduce only asexually, amongst the sexually reproducing species, 21 lack dwarf males and 25 are reported as nannandric, only two of which, *Dicranum scoparium* Hedw. and *Dicranum bonjeanii* De Not., have facultative dwarfism (Pichonet and Gradstein, 2012). In this study we focus on the widespread species *D. scoparium* sampled from seven localities, using 90 single-nucleotide polymorphic markers (SNPs). Our overall aims are to employ the molecular markers in a moss with facultative nannandry in order to (1) reveal the hierarchical genetic population structure (2) determine if the levels of genetic diversity and clonality compare between females and males and (3) assess the relative shares of dwarf males and normal-sized males to the total standing genetic diversity.

## Materials and Methods

### Study Species

*Dicranum scoparium* is widely distributed in the Holarctic and is growing on various substrates, preferably on acidic soil, rotten logs, tree stems and sometimes in sympatry with other *Dicranum* species (Crum and Anderson, 1981; Hedenäs and Bisang, 2004; Ireland, 2007; Lang et al., 2014). Both normal-sized and dwarf males can occur simultaneously in a same cushion. Dwarf males are annual and are easily found in the late summer on the tomentum of a female stem that carries a young sporophyte. They are supposedly non-clonal, whereas the perennial normal-sized males are clonal, resembling the females and, if present, found in the external part of a cushion.

### Sample Collection

**FIGURE 1:**
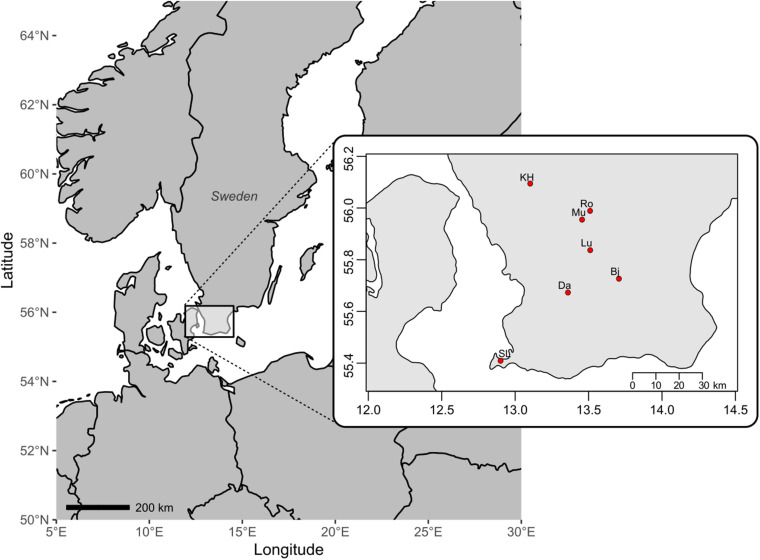
Map of southern Sweden with seven localities where populations of the moss *Dicranum scoparium* have been sampled: Bj, Bjärsjölagård; Da, Dalby; KH, Klöva Haller; Lu, Snogeröd Lunnerna; Mu, Munkarp; Ro, Röan; SL, Skanörs Ljung.

Fresh bryophyte material was collected in August 2015 across Skåne, South Sweden. In total 54 fertile cushions (including two cushions of *D. bonjeanii* and five of *Dicranum majus*) from 11 sites were collected from seven different localities, namely Snogeröd Lunnerna (Lu), Röan (Ro), Munkarp (Mu), Klöva Haller (KH), Skanörs Ljung (SL), Dalby (Da), Bjärsjölagård (Bj; Figure 1). In each of these localities, between one and three sites (if possible) were sampled. Two sites were considered distinct if they were separated from at least 2 km and by a river, road or ravine. Sexually reproducing populations are easily recognized in the field due to the presence of sporophytes and are generally associated with the presence of dwarf males and/or normal-sized males (Pichonet and Gradstein, 2012, pers. observ.). To perform genetic diversity analyses, we collected about 3 cm^2^ of *Dicranum* moss from sexually reproducing cushion. From each of these cushions, five fertile female stems were sampled. A stem can have several branches. Each of these branches were extracted separately as they also carried distinct sporophytes. Therefore, the number of female samples genotyped and then analyzed is bigger than five. All dwarf males found of on theses female stems were separated for *in vitro* growth and all normal-sized males within each of these cushions were sampled. The collected material was kept alive in the experimental garden of Lund University for morphological identification, resampling (if necessary), and further observation of vegetative growth.

### *In vitro* Culture of Dwarf Males

In order to get sufficient amounts of DNA or RNA from a single dwarf male individual we induced vegetative growth by separating them from the female and placing them onto a nutrient agar medium. Here, we used a medium as described in Rudolph et al. (1988). Each male was carefully removed from the female tomentum using a dissecting microscope and thoroughly inspected for contaminants before plating. The number of males found in each locality varied greatly, from only three males found in one cushion from Lunnerna to 384 found among the cushions from Bjärsjölagård. In order to remove bacterial, fungal or algal contamination, clean parts of the newly grown gametophyte were replated in new petri dishes regularly during a year after cleaning treatment. This treatment involved soaking the gametophyte in a solution of 0.05% NADCC at constant shaking for 1 min followed by two rinses, each 1 min in demineralized water. Removal of contamination remained the most difficult problem because males did not always survive the replating process. For example, 92 out of 526 dwarf males from Bj and only 2 out of 51 DM from Da survived. Hence, out of more than 500 dwarf males initially cultured, only 140 dwarf males were subject to DNA extraction (Supplementary Appendix 1).

### DNA Extraction

Total DNA from 769 samples including females, sporophytes, dwarf, and normal-sized males was extracted. Prior to extraction, stems of females, and normal-sized males were cleaned in demineralized water. After removing the surplus water with a tissue, all stems, sporophytes were placed in separate microtubes and kept in a −80°C freezer until extraction. After cultivation, dwarf males and associated protonemas were removed from the agar plates and dried in silica gel until extraction. DNA extractions were done using DNeasy Plant mini kit (Qiagen, Hilden, Germany) according to the protocol of the manufacturer and eluted in 100 μl AE buffer. All DNA extracts were stored at −20°C.

### RNA Extraction

We applied a strategy involving transcriptome analyses for selection of SNP markers, which requires RNA extracts of high quality and quantity. Fresh plant material was used for RNA extraction and one female stem per locality was randomly sampled in order to represent the genetic variability of the population. Three additional extraction was done for pooled normal-sized males, sporophytes (diploid generation) and one *Dicranum majus* Turner female gametophyte. Each stem was first cleaned in deionized water and then flash frozen in liquid nitrogen before being stored at −80°C. Consequently, RNA from ten samples were obtained using the RNeasy Plant mini kit following the protocol of the manufacturer (Qiagen, Hilden, Germany). The quality, quantity and integrity of the RNA extractions were measured on agarose gels, with Qubit^®^ 2.0 fluorometer (Thermo Fisher Scientific, Waltham, MA, United States) and BioAnalyzer (Agilent Technologies, Palo Alto, CA, United States), respectively.

### SNP Localization, Typification and Genotyping

The SNP library was produced with the MiSeq system (Illumina, United States) using the 300 bp paired-end settings. The library construction was carried out at Lund University sequencing facility^1^ using NEXTflex RNA-Seq kit (Bioo Scientific, Austin, TX, United States).

The sequencing of the moss transcriptomes resulted in about 1 million reads/sample (Supplementary Appendix 2). The sample DaF was used to create a *De Novo* reference assembly using Trinity (Grabherr et al., 2011; Haas et al., 2013). Reads from other samples were realigned using BOWTIE (Langmead et al., 2009) with –m option, allowing no multialigned reads in order to reduce as much as possible false positive SNP calling. We use the samtools (Li et al., 2009) mpileup tool to produce VCF files with all single nucleotide variants in the aligned BAM files. In total, nearly 25,000 variants were found. SNP filtering was done using scripts specifically designed for this purpose^2^. To remove variants called due to indel alignments, we removed SNPs within 100 bp of each other. To further support the authenticity of the variants, we selected only variants which were identified in at least three individuals. This also removes rare variants which are not useful in a genetic diversity screening (most variants are rare variants). Finally, to ensure the variants were still adequate markers of genetic diversity, we removed variants which were present in more than 8 (out of 10) samples. This resulted in a final selection of 119 SNPs.

The genotyping of 738 DNA samples was carried out at the SNP&SEQ Technology Platform, Department of Medical Sciences of Uppsala University. It was performed using a multiplexed primer extension chemistry of the iPLEX assay (Ross et al., 1998; Storm et al., 2003; Gabriel et al., 2009). The allele was then detected by mass spectrometry with a MassARRAY analyzer (Agena Bioscience, Hamburg, Germany). Raw data was converted to genotype data using Typer software (Agena Bioscience).

### SNP Data Analyses

The analyses were performed on *D. scoparium* individuals only, excluding sporophytes data. The final number of individuals analyzed in this study is found in Table 1. In order to analyze female and male genotypic diversity, or haplotypic diversity in this case, as well as levels of clonality within populations, we selected all gametophytic data from the raw SNP dataset (GAM). Then, because genet recruitment of female, normal-sized males, and nannandrous males was expected to follow different recruitment models, two subsets were created according to the sex of the samples, i.e., one subset containing female data (FEM) and one with male data (males). Additionally, to estimate the genetic differentiation within the two male types, dwarf males (DMs) and normal-sized males (NMs) were also analyzed separately. All analyses were performed in R (R Development Core Team, 2013) and genetic diversity metrics as well as levels of clonality within each population were characterized using the packages *adegenet* version 2.1.2 (Jombart, 2008; Jombart and Ahmed, 2011), *poppr* v. 2.8.5 (Kamvar et al., 2014, 2015), and *ade4 v. 1.7-15* (Chessel et al., 2004; Dray and Dufour, 2007; Dray et al., 2007; Bougeard and Dray, 2018). The analyses were performed with population hierarchy that included two levels, i.e., locality and cushion. Implementation and interpretation of *poppr* outputs were adapted from http://grunwaldlab.github.io/Population_Genetics_ in_R/ (Grünwald et al., 2017).

**TABLE 1:** Summary of the *D. scoparium* dataset showing sample size, number of haplotypes and codominant loci for each data subset after passing all filtering criteria.

Dataset	*n*	Loci	MLH	MLL
GAM	403	68	258	148
FEM	211	64	107	61
males	192	74	148	90
DM	134	74	113	72
NM	57	61	25	14

*Ten percentage or more missing data as well as invariable and uninformative loci were excluded for each data set independently. n, number of individuals; Loci, number loci; MLH, number of multilocus haplotype; MLL, number of multilocus lineages obtained after collapsing of unique MLH. Numbers are given for the total dataset (GAM) and the female (FEM), male (males) dwarf males (DM), and normal sized (NM) subsets.*

Firstly, failed genotyped individuals and SNPs were excluded from the raw data set. Then, the different subsets were created. Individuals and SNPs containing more than 10% missing information, invariable and uninformative loci (minimum allele frequency of 0.01) independently for each subset were excluded. Finally, we controlled again that the data set would contain no more than 10% missing information and eventually remove the individual or SNP that would not meet the criteria.

To remove the effect of genetic linkage due to clones and adjust for missing data and genotyping errors that may occur during sequencing large datasets (c.f. Kamvar et al., 2015), multilocus lineages (MLL) were calculated with the *mlg.filter* function by collapsing unique MLH (multilocus haplotypes) utilizing Nei genetic distance given the farthest neighbor clustering algorithm. This method allows to incorporate genotypes that have missing data or genotyping error into a parent clusters. The clustering threshold was set for each dataset individually and missing data were replaced with the mean of the alleles for the entire data set.

Multilocus lineages were further used for the estimation of the haplotypic diversity (G; Stoddart and Taylor, 1988) and Nei’s gene diversity, also called unbiased gene diversity (ĥ; cf. Grünwald and Hoheisel, 2006). Furthermore, the relative contribution of clonality within a population was assessed by using the index of association (IA) and its standardized form, r̄_*d*_, which accounts for the number of loci sampled (Agapow and Burt, 2001). Considering that IA is based on the variance of pairwise distances between MLLs, IA should be high in clonal populations and low in populations undergoing sexual reproduction (Grünwald and Hoheisel, 2006). A significance test with 1000 permutations was run to assess for significance. Hierarchical structure of genetic variation was explored by running an analysis of molecular variance [AMOVA (Excoffier et al., 1992)] considering cushion as subpopulations within localities. The analysis was conducted using the *poppr.amova* function and the *ade4* implementation. Fixation indices (Φ statistics) were interpreted as a measure of subpopulation differentiation. The probability of retrieving the observed Φ under the null hypothesis of no differentiation between subpopulations was calculated using the *randtest* function of the *ade4* package with 1000 iterations of the permutation test.

Assuming that the diversity of DM haplotypes residing on a female is determined by the number male spores that germinated on the female, the proportion of DMs should covary with haplotypic diversity in a population. Therefore, the proportion of each sex type at locality was plotted against the haplotypic diversity G. Kruskal Wallis non-parametric test was used to examine the differences in G between sex types. Furthermore, an ANOVA was used to test for the differences of ĥ between sex types as residuals followed a normal distribution. We also compared Nei’s gene diversity ĥ against G in order to display the contribution of each haplotype to the total variability at cushion level.

Finally, population structure was analyzed in two ways. First, a principal coordinate analysis (PCoA) was performed on female and male individuals. The analysis was based on a pairwise genetic distance matrix, with Prevosti’s distance, equivalent to dissimilarity distance. Then, a minimum-length spanning network (msn) of all unique MLL was constructed based on the Prevosti’s distance in order to assess how female and male haplotypes were related across different populations. Resulting graphs were edited with Inkscape v.1.0.0.

## Results

### SNP Detection in RNA and DNA Genotypification

From the 10 initial samples, 119 SNPs were identified for genotype screening (see section “Materials and Methods”). Out of 769 extracted DNA samples, 738 were suitable for genotyping, whereas 31 lacked enough DNA for genotyping. Of the 119 SNPs, 90 SNP markers were identified in all 738 genotyped DNA samples. The fraction of SNP markers with call rate >0 reached 75.63% and the average sample call rate per SNP reached 88.49%. The fraction of individuals with call rate >0 reached 95.80% while the average SNP call rate per sample reached 69.86%. Five out of 90 SNPs were invariable (5.56%). The 29 deficient SNPs had no allele signal cluster separation (sample call rate of 0%) which resulted in inaccurate genotype calls.

### Higher Genetic Diversity Among Females and Dwarf Males Than Among Normal-Sized Males

After exclusion of individuals and SNPs with 10% or more missing data and invariable loci, the total *D. scoparium* haploid dataset contained 403 individuals and 68 loci (Table 1). The proportions of males (NMs and DMs) and females reflect the frequencies at each site. Five sites had both NMs and DMs, three had only NMs and two only DMs (Table 2). The number of DMs differed strongly between sites, and when present, they usually outnumbered both females and NMs, and at one site (Bj), they were particularly abundant (Table 2). The female dataset was composed of 211 individuals and 64 loci, representing 61 MLLs. The male dataset contained 192 individuals and 74 loci, representing 90 MLLs (Table 1).

**TABLE 2:** Number of *D. scoparium* individuals in each data subset used for the analysis after passing all filtering criteria.

Locality	Site	Cushion	FEM	DM	NM
Bj	1	3	21 (2)	88 (43)	0
Da	1	4	19 (5)	2 (2)	2 (1)
KH	1	4	18 (8)	0	10 (2)
KH	2	6	15 (6)	0	1 (1)
Lu	1	3	7 (4)	3 (1)	2 (1)
Lu	2	1	5 (1)	0	0
Lu	3	2	9 (6)	0	2 (2)
Mu	1	8	30 (9)	4 (3)	8 (3)
Ro	1	5	23 (6)	25 (17)	8 (2)
Ro	2	5	18 (6)	6 (2)	0
SL	1	6	46 (9)	6 (5)	24 (3)
Total	16	47	211 (61)	134 (72)	57 (15)

*The localities are Bj, Bjärsjölagård; Da, Dalby; KH, Klöva Haller; Lu, Snogeröd Lunnerna; Mu, Munkarp; Ro, Röan; SL, Skanörs Ljung. Numbers in parenthesis are the number of unique multilocus lineage, corresponding to the number of individuals with unique haplotype. Site, site number within the locality; Cushion, number of cushions within each site; FEM, number of females; DM, number of dwarf males; NM, number of normal-sized males.*

**FIGURE 2:**
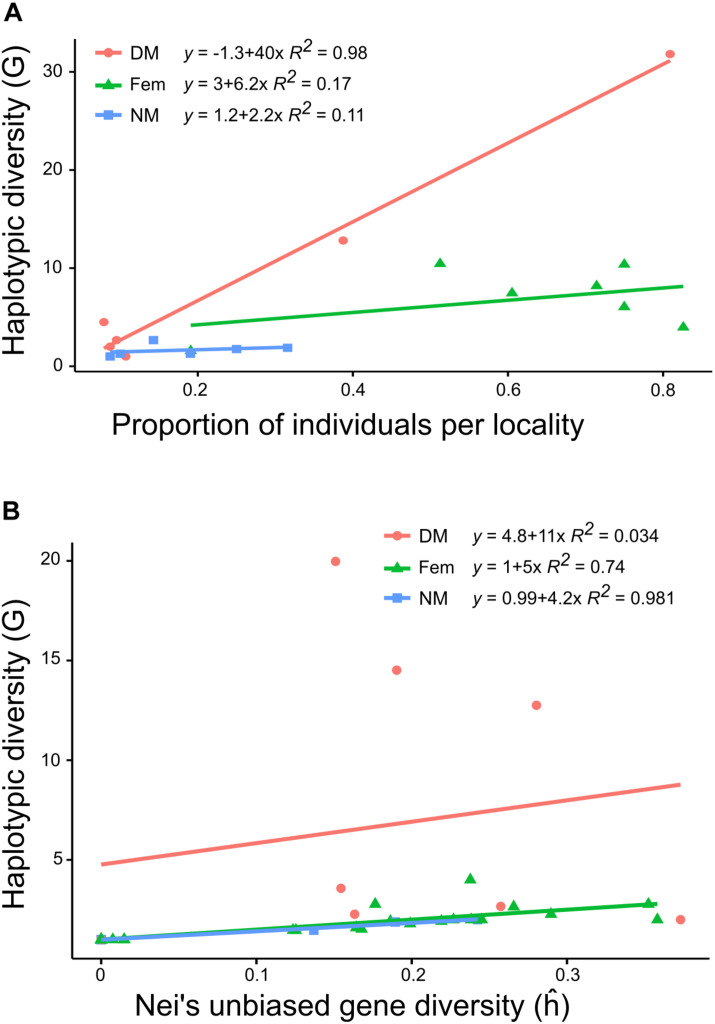
Scatter plot with regression lines and R^2^ showing **(A)** the proportion of individuals at locality level against the genotypic diversity (G) and **(B)** the expected gene diversity (ĥ) against G for each sex type (dwarf males, females, and normal-sized males). Colors of regression line and point represent sex types (red dot, dwarf males; Green triangle, females; Blue square, normal-sized males).

Multilocus haplotypic diversity (G) differed between localities, from *G* = 5.45 in Dalby to *G* = 18.39 in Bjärsjölagård and the underlying proportions of female, NM and DM haplotypes were profoundly disparate. The NMs had significantly lower haplotypic diversity (mean *G* = 1.8) than both females (mean *G* = 6.9) and DMs (mean *G* = 8.2) (Kruskal–Wallis; *H* = 6.8; df = 2; *P* = 0.03). Furthermore, the haplotypic diversity for DMs varied a lot dependent on the number of individuals that were possible to genotype (mean *G* = 8.2; Table 3). Although the different sample sizes explain some of the variation in G, Figure 2A illustrates that the proportion of females or NMs in a population had low influence on haplotypic diversity and that only the proportion of DMs correlated with haplotypic diversity (*R*^2^ = 0.98), although this is largely due to two localities with abundant population of dwarf males (Bj and Ro).

**TABLE 3:** Genetic diversity at locality level for the combined dataset (GAM), female (FEM), dwarf male (DM), normal-sized male (NM), and all male (males) data subsets.

Locality		*n*	MLH	MLL	G	ĥ	IA	r̄_*d*_
Bj	GAM	110	80	49	18.39	0.19	8.44	0.18
	FEM	21	8	2	1.57	0.16	26.19	0.97
	males	89	74	49	32.07	0.19	6.8	0.13
	DM	88	76	43	26.34	0.2	7.61	0.14
	NM	0						
Da	GAM	23	15	8	5.45	0.31	6.78	0.14
	FEM	19	9	5	3.97	0.29	8.88	0.21
	males	4	4	3	2.67	0.31	8.77	0.22
	DM	2	2	2	2	0.4	NA	NA
	NM	2	2	1	1	0	NA	NA
KH	GAM	44	30	17	11.13	0.33	2.71	0.04
	FEM	33	18	14	10.37	0.33	2.46	0.04
	males	11	7	3	1.75	0.17	20.14	0.52
	DM	0						
	NM	11	5	3	1.75	0.19	19.4	0.53
Lu	GAM	28	17	13	7.84	0.34	7.03	0.13
	FEM	21	11	10	6.04	0.34	7.59	0.15
	males	7	5	3	1.81	0.21	22.65	0.6
	DM	3	2	1	1	0	NA	NA
	NM	4	4	3	2.67	0.35	11.57	0.33
Mu	GAM	42	30	14	10.63	0.32	3.4	0.06
	FEM	30	21	9	8.18	0.31	3.45	0.07
	males	12	9	5	2.57	0.2	14.12	0.29
	DM	4	4	3	2.67	0.28	17.47	0.47
	NM	8	3	3	1.68	0.09	19.16	0.96
Ro	GAM	80	51	32	19.05	0.36	2.68	0.05
	FEM	41	24	12	10.44	0.35	3.1	0.06
	males	39	28	21	12.17	0.32	4.27	0.07
	DM	31	23	19	12.81	0.32	5.21	0.09
	NM	8	3	2	1.28	0.08	18.82	0.99
SL	GAM	76	35	16	9.66	0.35	4.56	0.07
	FEM	46	16	9	7.45	0.37	5.43	0.09
	males	30	21	7	2.87	0.24	14.06	0.3
	DM	6	6	5	4.5	0.24	9.38	0.24
	NM	24	8	3	2.27	0.18	21.13	0.92
Average	GAM				11.74	0.31		
	FEM				6.86	0.31		
	males				7.99	0.23		
	DM				7.05	0.24		
	NM				1.68	0.15		

*n, sample size; MLH, Multilocus haplotype; MLL, Multilocus lineages; G, Haplotypic diversity based on Stoddard and Taylor’s index; ĥ, Nei’s unbiased gene diversity; IA, index of association; r̄_*d*_, Standardized IA. All ĥ and IA values were significant (*P* < 0.001), based on 1,000 permutations.*

Overall, levels of gene diversity ĥ were slightly higher for females (mean ĥ = 0.31) than males (mean ĥ = 0.23) (ANOVA; *F* = 4.83; *P* = 0.048). The DMs (mean ĥ = 0.24) were usually more variable than the NMs (mean ĥ = 0.15) and NMs had significantly lower gene diversity than both females and DMs (ANOVA; *F* = 4.22; *P* = 0.03). In most localities NMs had ĥ values ranging between 0 and 0.19, but one exception was found at Lu, with ĥ = 0.34 for both females and NMs (Table 3). At cushion level, which is the scale relevant for fertilization, gene diversity was strongly positively correlated with haplotypic diversity, except at patches with numerous DMs (Figure 2B). This means that each normal-sized haplotype contributes about equally much to the total genetic variability at cushion level. The cushion level estimate of ĥ is equal or higher for DMs as compared to NMs, but the variation is potentially distributed among many more haplotypes for DMs.

The degree of clonality was measured with the index of association (IA). Clonality was strongest for NMs at all localities, with high IA values and r̄_*d*_ values ranging between 0.32 and 1, suggesting high clonality. As expected, DMs had r̄_*d*_ values close to 0, which indicate low clonality but somewhat surprisingly, female IA and r̄_*d*_ values were similar to those obtained for DMs. The populations in Bj showed an opposite pattern: levels of haplotypic diversity were very low for females and very high for males, although levels of gene diversity were comparable. Furthermore, IA and r̄_*d*_ values indicated high clonality of females at this locality.

### Population Structure

Almost 90% of the total variation was partitioned nearly equally within cushions and among cushions within localities, while only 11% of the variation occurred among localities (Table 4: GAM). 25% of the molecular variance was associated with a differentiation within female cushions (haplotypes among localities) and 68% with differentiation among cushions within localities (haplotypes among cushions within localities). Males also displayed a high relative differentiation at cushion level (DM = 47.1% and NM = 30%, respectively). The highest fixation index was within cushions in both females and NMs (FEM: Φ = 0.75; NM: Φ = 0.7).

**TABLE 4:** Analysis of molecular variance (AMOVA).

Dataset	Hierarchical level	Df	Sum of squares	Mean squares	Variation (%)	Φ	*P*
GAM	Φ within cushion	356	3886.05	10.92	42.59	0.57	*0.001*
	Φ among cushion within locality	40	3837.14	95.93	45.92	0.52	*0.001*
	Φ among locality	6	2110.06	351.68	11.48	0.11	*0.001*
FEM	Φ within cushion	164	1022.13	6.23	25.3	0.75	*0.001*
	Φ among cushion within locality	40	3130.33	78.26	68.27	0.73	*0.001*
	Φ among locality	6	890.44	148.41	6.43	0.06	*0.001*
DM	Φ within cushion	123	1637.63	13.31	47.14	0.53	*0.001*
	Φ among cushion within locality	5	386.62	77.32	19.8	0.3	*0.001*
	Φ among locality	5	950.75	190.15	33.06	0.33	*0.040*
NM	Φ within cushion	46	338.72	7.36	30.01	0.7	*0.001*
	Φ among cushion within locality	5	145.02	29	50.01	0.62	*0.003*
	Φ among locality	5	667.47	133.49	19.98	0.2	*0.004*

*AMOVA results for combined data (GAM), female (FEM), dwarf males (DM), and normal-sized males (NM). *P* values were obtained for the Φ statistic with 1000 permutations.*

**FIGURE 3:**
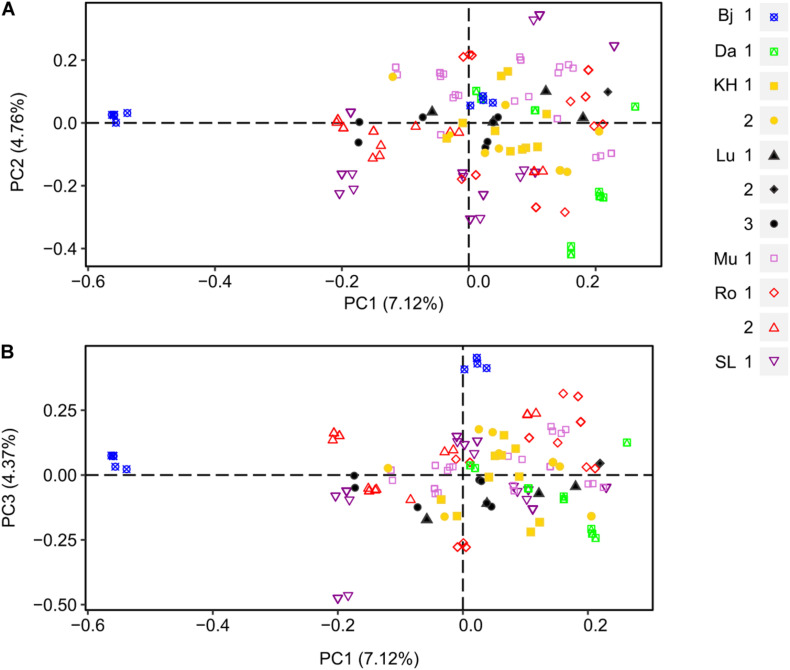
Principal Coordinate Analysis (PCoA) of female populations of *D. scoparium* from southern Sweden based on a genetic distance matrix. Individuals are represented by a symbol according to the locality (Bj, blue; Da, green; KH, yellow; Lu, black; Mu, light purple; Ro, red; SL, purple) and the collection site (number 1.2 or 3). The axis 1 and 2 are shown in scatter plot **(A)** and axis 1 and 3 in scatter plot **(B)**.

A PCoA was produced to visualize the genetic distance of female and male individuals (Figure 3). Overall, the low differentiation among the female samples at locality levels observed in the AMOVA analyses was reflected in the PCoAs. The first three axes explained 16.25% of cumulative variation (PC1: 7.12%, PC2: 4.76%, and PC3: 4.37%). No clear subclustering was displayed, only few individuals from Bj were forming a separate cluster, best separated by PC1 and PC3. However, looking at locality with more than one site individually (Lu, Ro), some fine structure could be seen. In population Lu (black shapes), site 1 and 3 were forming two clusters with certain degree of overlapping and site 2 was more distant from the two former. Ro had two sites (red empty shapes) which were separated by PC1 and PC2. Although SL had only one site, the individuals were forming two clusters separated by PC2.

**FIGURE 4:**
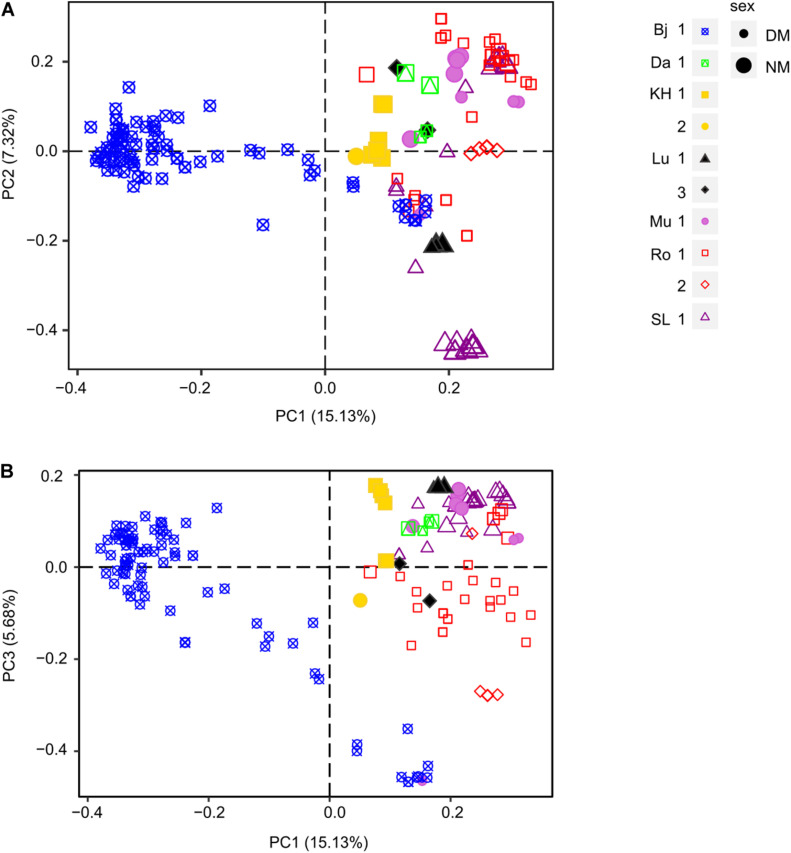
Principal Coordinate Analysis (PCoA) of male populations of *D. scoparium* from southern Sweden based on a genetic distance matrix. Individuals are represented by a symbol (large, NM; small, DM) according to the locality (Bj, blue; Da, green; KH, yellow; Lu, black; Mu, light purple; Ro, red; SL, purple) and the collection site (number 1.2 or 3). The axis 1 and 2 are shown in scatter plot **(A)** and axis 1 and 3 in scatter plot **(B)**.

The first three axes of the male PCoA explained 28.13% cumulative variance (PC1: 15.13%, PC2: 7.32%, and PC3: 5.68%). Similar to the females, the male PCoA displayed little structure at locality level (Figure 4), except for Bj. Most individuals (all dwarf males) of this locality were separated by PC1 from all other individuals, all localities included. Whereas no differentiation could be revealed at site level, more structure was observed at the sex level, i.e., between normal-sized males (NM, big shapes) and dwarf males (DM, small shapes). NMs from a certain site tended to cluster more closely, being more similar to each other than DMs. PC2 segregated strongly the individuals in SL 1 into three clusters, two containing only NMs and one containing only DMs (purple triangles). Furthermore, individuals from Lu 1 (black triangles) were strongly separated from the individuals from Lu 3. Finally, PC3 tended to segregate individuals from KH 1 and 2, Lu 1 and 3 as well as DMs and NMs from Ro 1.

**FIGURE 5:**
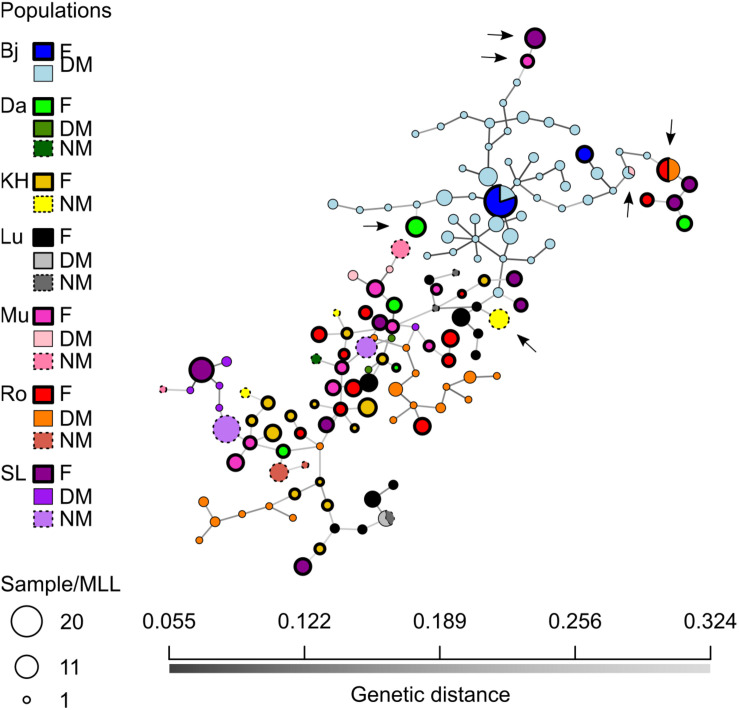
Minimum-spanning network (MSN) of the *Dicranum scoparium* SNPs. Each node represent a unique multilocus lineage (or haplotype in our case). Colors represent sexes per localities and edges represent absolute genetic distance (Bj, blue; Da, green; KH, yellow; Lu, black; Mu, pink; Ro, red; SL, purple; F, female; DM, dwarf-male and NM, normal-sized male).

To visualize relationships between female and male MLLs, a minimum-spanning network was constructed (Figure 5). The number of individuals per MLL (or haplotype) ranged between 1 and 16. Sixty-one female MLLs, 13 NM MLLs, and 77 DM MLLs were having more than one individual of which 7, 1, and 2 MLLs for female, NM and DM, respectively, where shared between individual of different cushions. Additionally, two genotypes were shared between females and DMs, one genotype was shared between DM and NM and one genotype was shared between DM across localities (arrows in Figure 5). This last genotype was found in two individuals of population Bj and one of Mu. Overall, there was no clear differentiation of the populations, apart from population Bj. In this populations, DM genotypes were much closer to each other than to genotypes of other populations. Furthermore, one of the two female genotypes was shared among 20 individuals, sampled in two different cushions and both sexes.

## Discussion

In this study, we examined the genetic variability and structure of female and male populations of the facultatively nannandric moss *Dicranum scoparium* at different scales. Dwarf males and normal-sized males often occurred together in a moss cushion. We showed that the overall levels of genetic diversity within females and dwarf males were relatively similar and much higher than among normal-sized males. The lower genetic diversity in NMs may therefore be a consequence of fewer haplotypes in the populations. Furthermore, clonality occurred in all three categories, yet at different degrees: level of clonality was high among NMs whereas it was much less so in females and DMs. Most of the genetic differentiation was partitioned within and among cushions and only a low fraction among localities. These results suggest that effective gene flow occurs regularly over long distances.

### Experimental Design

Before we interpret the genetic diversity data, it is worth mentioning that (1) we have actively searched for loci that are variable and (2) that the regions are transcribed and therefore the SNP alleles are possibly under selection. This implies that absolute values of genetic diversity measures are not directly comparable with other studies, although our markers in several ways (representing expressed variation, point mutations) resemble allozyme markers, for which much of the population level studies were done in the past.

No reference genome for *D. scoparium* exists. Thus, designing a genetic diversity panel is challenging. Our approach, using RNA-seq and transcriptome assembly enables an affordable construction of transcribed portions of the genome that are likely evolutionarily relevant. Yet, the quality of such an assembly is highly dependent on the depth of sequencing, which is highly variable per transcript, and especially near the end of transcript. Furthermore, SNPs were identified using RNA-Seq reads aligned to the assembled transcriptome. This too is restricting and resulted in only 119 reliable SNPs. Then, due to the cost of RNA-Sequencing, we used only 10 samples (one per locality) that would represent the total genetic diversity of each population. Hence, we selected variants that were relatively common and not entirely recurrent (in at least three and at most eight of the 10 samples). Furthermore, the extraction and genotyping steps themselves also lead to some losses. For example, DM for the populations in Lu were extracted but the genotyping was not successful. Finally, SNP data pose new challenges when compared to other markers, because they potentially contain more missing data, error in SNP calling due to genotyping errors or other sources of inaccurate allele calls (Mastretta-Yanes et al., 2015). Having this in mind, we note that our data set produced clear results with respect to clonal diversity, gene diversity and genetic population structures for the whole material and when separated into NMs, DMs and females.

### Genetic Diversity and Population Structure

Genetic diversity of females and all males taken together were similar, but NMs tended to be genetically less diverse relative to DMs, while females and DMs had comparable levels of genetic diversity. The PCoA, MSN, and AMOVA analyses showed weak genetic differentiation at larger scale, except for the locality Bjärsjölagård (Bj). At locality levels genetic differentiation for females was particularly low (Φ = 0.06), which is indicative of a high gene flow. The corresponding values for *H. lutescens* at locality level was around 0.10 (Rosengren et al., 2013) and 0.3 (Rosengren et al., 2015) from different regions in S Sweden (Öland and Scania, respectively), suggesting a somewhat higher differentiation among those populations even though the sampling of *D. scoparium* spanned a larger geographic area. The difference between these two nannandric species could potentially be explained by the degree of isolation between populations and/or the fact that perennial, normal-sized males are much rarer in *H. lutescens*, increasing the risk for inbreeding due to son-mother fertilization with DMs.

A large fraction of the total variation in our study partitioned within cushions and among cushions within sites, suggesting that cushions in general have several genets which are notably dissimilar. This is particularly valid among DMs, where the fraction of the total variation residing within cushions reached 47%. Our findings indicate that gene flow occurs between localities and that the relatively high levels of genetic diversity, both within females and males, are maintained through spore dispersal rather than vegetative fragment dispersal. The structure found in *Dicranum* populations are consistent with other moss studies that indicated limited population structure at larger scales but differentiation at cushion level (Cronberg, 2002; McDaniel and Shaw, 2005; Hutsemékers et al., 2010; Paasch et al., 2015; Désamoré et al., 2016). DMs and NMs often occurred simultaneously but the two types of males are obviously under different constraints and thereby contribute differently to the population dynamics. When present, dwarf males are often numerous and display a level of genetic diversity similar to the one of females. The proportion of DMs covaries positively with the haplotypic diversity of a population (at locality level), which is not the case for females and NMs. The vicinity of DMs to the female perichaetia is likely to be a great advantage in competition with NMs for fertilization, but NMs may be important for fertilization when conditions are unfavorable for DMs, as they are perennial and probably less sensitive to temporarily adverse climatic conditions. In this context is worth to notice that, if anything, the haplotypic and genetic diversity of DMs may be underestimated since a high number of the DMs were lost before DNA extraction.

The level of gene diversity found in *D. scoparium* is comparable to the dwarf males and females of the nannandric species *H. lutescens* (Rosengren et al., 2013, 2016). Although males were most likely dispersed locally, the authors showed that a fraction of males originated from more distant populations, hence maintaining high levels of genotypic diversity within cushions and across localities. In our study, we found NMs growing on soil, at the border of cushions, with generally much lower haplotypic diversity than DMs and females. This compares with results obtained for the non-nannandric species *Syntrichia caninervis* (Baughman et al., 2017), whose males displayed a lower genetic diversity than females. In both *H. lutescens* and *S. caninervis*, the differences in diversity within the male and female populations can be attributed to local environmental conditions such as moisture and temperature, which may affect spore dispersal and germination success (McDaniel et al., 2007; Rosengren and Cronberg, 2014; Rosengren et al., 2016; Baughman et al., 2017). Whereas several studies have demonstrated the positive association between moisture and presence of males (Tamm, 1964; Bowker et al., 2000; Sagmo Solli et al., 2000; Rosengren and Cronberg, 2014), the environmental constraints on NMs are largely unknown.

Finally, the haplotype networks provided some evidence of inbreeding events occurring in populations, especially at Ro and Bj. Whereas extreme inbreeding indicative of repeated son-mother fertilizations was fairly frequently found in *H. lutescens* (Rosengren et al., 2016), only two haplotypes were shared between females and DMs in our study (Figure 5). However, the DM haplotypes were sometimes clustering tightly together with the female haplotype they were growing on as seen in the network analysis (Figure 4). Therefore, repeated son-mother fertilizations may occur in *D. scoparium* as well, but in general, external DM recruitment seems common enough to prevent extensive inbreeding.

### Clonal Diversity

Although the scale of the sampling was not designed to elucidate the clonal structure of populations, we observed that the overall levels of clonality was low and that there was not much difference between the sexes, i.e., females and both types of males taken together. When separating the data set into DMs and NMs, it appears that the male clonality estimate is strongly influenced by DMs, lowering the index of association r̄_*d*_. Indeed, we found a higher degree of clonality in NMs as compared to DMs and females, with r̄_*d*_ values close to 1 (Table 3), suggesting more extensive clonal propagation for NMs and values closer to zero for DMs, indicating recombination. Previous studies on the perennial pleurocarps *Hylocomium splendens* and *H. lutescens* have brought out that populations often contains multiple, but few, genets in patches, suggesting that clonal expansion predominates over sexual recruitment (Cronberg et al., 2006b; Rosengren et al., 2013). Our results indicate that sexual reproduction in the studied populations more often results in recruitment of new female genets, also within female colonies. A high genotypic female diversity at patch level could possibly be explained by imperfect female control of spore germination, so that new normal-sized females sometimes are recruited along with the DMs. Rarer occurrence of NMs and their peripheral distribution in patches, indicate higher male mortality rates at recruitment and/or that female-dominated colonies forces male spores to become DMs and prevent them from escaping dwarfism by falling off the female, at least at the colony center. In order to better explain the control of development into NMs and DMs and associated patterns of genetic variability, further experimental studies are needed.

### Bjärsjölagård (Bj): The Rise of a New Population?

The population of Bjärsjölagård (Bj) differs in several aspects; it is genetically divergent despite being geographically relatively close to sites Lu and Da (Figure 1). It probably represents a recent colonization event followed by external male recruitment, which may spread some light on genetic consequences of a founder event in a species with nannandry. The site is located in a former lime quarry, which is not a normal substrate for *D. scoparium* to occur, having preference for acidic soils at a pH around 3.2–7.1 (Tyler and Olsson, 2016). The previously open and barren calcareous ground has been partially covered by detritus from a scattered canopy of colonizing *Fagus sylvatica*, *Corylus avellana*, and *Acer platanoides* trees, allowing *D. scoparium* to establish together with species such as *H. splendens* in competition with more calciphilous species. At this locality, we found three isolated female cushions with sporophytes separated by maximum 1 m. No NMs were found, but the population of DMs was the densest of our study, with up to 88 genotyped DMs (Table 2; pers. obs.). The haplotypic diversity of the total dataset (GAM) harbored at Bj was fairly similar to the one at Ro. Yet, gene diversity was the lowest of all the localities (ĥ = 0.19; Table 3). In further comparison to Ro, which had a similar number of females genotyped, we detected only two haplotypes within female populations. Consequently, they retained the lowest haplotype and gene diversity (*G* = 1.57 and ĥ = 0.16, respectively) as well the highest index of association (r̄_*d*_ = 0.97) among all female populations. These results indicate that vegetative growth of female cushions is predominant at this locality and are congruent with other moss species (Cronberg et al., 2006b; Rosengren et al., 2013; Baughman et al., 2017). Moreover, five female MLLs from other populations, namely from Mu, Lu, Ro, SL, and Da (arrows in Figure 5), were closer genetically to DMs of Bj than individuals of any other localities. Finally, two DMs shared the same MLL with one male from Mu, making it the only MLL shared across localities. These results provide evidence that spores are dispersed over long distances and that male spores germinate more easily on female stems than on soil. Thus, even if the colonizing female population was not diverse, the DM male recruitment via spores results in quite extensive recombination events, which lead to an increase in genotypic diversity.

### Conclusion

The genetic influence of dwarfism on population structure has rarely been studied and only few comparable studies exist. Here, we used SNP markers to investigate the genetic structure of seven populations of the facultatively nannandric species *D. scoparium* in Scania (Sweden) in a similar way as earlier done for the obligately nannandric species *H. lutescens*. The genetic differentiation at locality level was low indicating a strong gene flow. In the dense acrocarpous cushions of *D. scoparium*, the haplotypic diversity at patch level was higher for females (and NMs) than in the pleurocarpous and weft-forming *H. lutescens*, suggesting more frequent recruitment.

In *D. scoparium*, DMs and NMs often occurred together but the DMs were genotypically more frequent and divergent. The genotypic networks show that DM individuals at patch level represent both closely related haplotypes (suggesting inbreeding) and remotely related haplotypes (suggesting external gene flow). Nevertheless, NMs may regularly take part in fertilization as they are perennial and probably less sensitive to temporarily adverse climatic conditions. If so, the facultative nannandric system is prevented from transforming to obligate nannandry through sexual selection, contrasting to the nearly obligate or obligate nannandric conditions in *H. lutescens* and most *Dicranum* species.

Nannandry has probably developed to ensure fertilization and increase the number of male gamete donors. We see nannandry in mosses as a fertilization syndrome, a parallel to pollination syndromes in flowering plants, which ensure pollination and increases the available number of pollen donors. More studies on sexual reproduction and diversity in nannandric mosses are needed, especially in regards to facultative dwarfism. It would be particularly useful to determine the environmental condition for the recruitment and growth of normal-sized males to better understand their contribution to the population dynamics.

## Data Availability Statement

The raw dataset generated for this study can be found in the European Nucleotide Archive (https://www.ebi.ac.uk/ena/) under accessions PRJEB37280 and ERP119316 (secondary study accession).

## Author Contributions

AL and NC wrote the text, conducted the sampling of the specimens, and participated in the interpretation of the results. TG and NC commented and critically reviewed the manuscript. AL conducted the laboratory work and the data analyses. TG designed the scripts specifically designed for SNP filtering. All authors approved the final manuscript.

## Conflict of Interest

The authors declare that the research was conducted in the absence of any commercial or financial relationships that could be construed as a potential conflict of interest.

## Footnotes

1 https://www.biology.lu.se/services/dna-sequencing-facility/next-generation-sequencing-on-illumina-miseq

2 https://github.com/thiesgehrmann/annickMossVarCall

## Abbreviations

NMs: normal-sized males
DMs: dwarf males
Sampled localities in Southern Sweden: Lu: Snogeröd Lunnerna
Ro: Röan
Mu: Munkarp
KH: Klöva Haller
SL: Skanörs Ljung
Da: Dalby
Bj: Bjärsjölagård.
