# Supplementary material for: Genetic Diversity and Population Structure in Bryophyte With Facultative Nannandry
Source: Front Plant Sci. 2021 Apr 7;12:517547. doi: 10.3389/fpls.2021.517547 (PMC8059434; doi:10.3389/fpls.2021.517547)
Supplement: Supplementary file 1 [file Data_Sheet_1.DOCX]

**Appendix 1:** Number of *D. scoparium* individuals sampled in each site. The number of collected DMs and the number of individual for which DNA has been extracted for DMs, females and NMs is given.

| Locality | site | Collected DM | Extracted DM | Extracted FEM | Extracted NM |
| --- | --- | --- | --- | --- | --- |
| Bj | 1 | 526 | 92 | 24 | 0 |
| Da | 1 | 51 | 2 | 32 | 3 |
| KH | 1 | 0 | 0 | 25 | 10 |
| KH | 2 | 0 | 0 | 18 | 1 |
| Lu | 1 | 3 | 3 | 8 | 2 |
| Lu | 2 | 0 | 0 | 5 | 0 |
| Lu | 3 | 0 | 0 | 10 | 2 |
| Mu | 1 | 6 | 4 | 35 | 8 |
| Ro | 1 | 46 | 26 | 28 | 8 |
| Ro | 2 | 10 | 6 | 22 | 0 |
| SL | 1 | 16 | 7 | 55 | 34 |
| Total | 16 | 658 | 140 | 262 | 68 |

Collected DM, number of DM collected and put in culture; Extracted FEM, number of females corresponding to the number of sampled individuals; Extracted NM, number of NM corresponding to the number of sampled individuals

**Appendix 2:** Number of transcriptome reads. One female individual per locality (locality) as well as pooled males (M), diploid sporophytes (S) and one female *D. majus* sample (DmajF) have been used for RNA transcriptome sequencing and further SNP calling.

| locality | Nb raw reads | Pair-trimmed | Unpaired-trimmed (R1/R2) | Nb discarded reads |
| --- | --- | --- | --- | --- |
| BjF | 10785916 | 2092017 | 1098191/84392 | 7511316 |
| DaF | 14374188 | 3241174 | 967223/197543 | 9968248 |
| DmajF | 11150424 | 2201257 | 1177627/77767 | 7693773 |
| KHF | 16271812 | 3658210 | 1103765/215454 | 11294383 |
| LuF | 11965000 | 2747114 | 784948/163696 | 8269242 |
| M | 11682980 | 2640787 | 802520/171682 | 8067991 |
| MuF | 14784384 | 3384286 | 1010724/189490 | 10199884 |
| RoF | 14125172 | 3233670 | 952170/185108 | 9754224 |
| SLF | 12571152 | 2866014 | 824242/174011 | 8706885 |
| S | 12740704 | 2936551 | 851960/159461 | 8792732 |
